# Identifying optimal bioinformatics protocols for aerosol microbial community data

**DOI:** 10.7717/peerj.12065

**Published:** 2021-09-30

**Authors:** Katie Miaow, Donnabella Lacap-Bugler, Hannah L. Buckley

**Affiliations:** School of Science, Auckland University of Technology, Auckland, Auckland, New Zealand

**Keywords:** Bioaerosol, Bioinformatics, Microbial ecology, NGS, Bacteria, Fungi, Microbial aerosol, USEARCH, Decontamination, Dada2

## Abstract

Microbes are fundamental to Earth’s ecosystems, thus understanding ecosystem connectivity through microbial dispersal is key to predicting future ecosystem changes in a warming world. However, aerial microbial dispersal remains poorly understood. Few studies have been performed on bioaerosols (microorganisms and biological fragments suspended in the atmosphere), despite them harboring pathogens and allergens. Most environmental microbes grow poorly in culture, therefore molecular approaches are required to characterize aerial diversity. Bioinformatic tools are needed for processing the next generation sequencing (NGS) data generated from these molecular approaches; however, there are numerous options and choices in the process. These choices can markedly affect key aspects of the data output including relative abundances, diversity, and taxonomy. Bioaerosol samples have relatively little DNA, and often contain novel and proportionally high levels of contaminant organisms, that are difficult to identify. Therefore, bioinformatics choices are of crucial importance. A bioaerosol dataset for bacteria and fungi based on the 16S rRNA gene (16S) and internal transcribed spacer (ITS) DNA sequencing from parks in the metropolitan area of Auckland, Aotearoa New Zealand was used to develop a process for determining the bioinformatics pipeline that would maximize the data amount and quality generated. Two popular tools (Dada2 and USEARCH) were compared for amplicon sequence variant (ASV) inference and generation of an ASV table. A scorecard was created and used to assess multiple outputs and make systematic choices about the most suitable option. The read number and ASVs were assessed, alpha diversity was calculated (Hill numbers), beta diversity (Bray–Curtis distances), differential abundance by site and consistency of ASVs were considered. USEARCH was selected, due to higher consistency in ASVs identified and greater read counts. Taxonomic assignment is highly dependent on the taxonomic database used. Two popular taxonomy databases were compared in terms of number and confidence of assignments, and a combined approach developed that uses information in both databases to maximize the number and confidence of taxonomic assignments. This approach increased the assignment rate by 12–15%, depending on amplicon and the overall assignment was 77% for bacteria and 47% for fungi. Assessment of decontamination using “decontam” and “microDecon” was performed, based on review of ASVs identified as contaminants by each and consideration of the probability of them being legitimate members of the bioaerosol community. For this example, “microDecon’s” subtraction approach for removing background contamination was selected. This study demonstrates a systematic approach to determining the optimal bioinformatics pipeline using a multi-criteria scorecard for microbial bioaerosol data. Example code in the R environment for this data processing pipeline is provided.

## Introduction

Next generation sequencing (NGS) of microbial marker genes has revolutionized microbiome studies (Pearce et al., 2016). Modern techniques are extremely sensitive, and can detect even one copy of a target gene (McKnight et al., 2019). Most environmental microorganisms grow poorly in culture and can be present in very small numbers (Burrows et al., 2009). NGS circumvents these issues, as different genetic variants present can be inferred, and their taxonomy predicted. The microbiome of the aerosphere is challenging to decode, even with these new approaches. It is a very low biomass environment, and many common contaminants (microbial taxa that are not true constituents of the environmental population) are naturally present in air. Indeed, these microbes are probably contaminants themselves due to their ease of atmospheric dispersal. Study of the aero-microbiome is relatively recent, and unknown taxa are frequently detected (Bottos et al., 2014). The low biomass and many ubiquitous taxa present make identification and removal of contamination difficult. While biomass can be improved with greater sample durations (Pearce et al., 2009), DNA degradation and practical considerations can preclude prolonged sampling campaigns (Luhung et al., 2015). Further, it is challenging to tell which taxa represent true variants and to assign taxonomy to them with existing databases. Due to these difficulties, single amplicon sequencing (commonly 16S, 18S ribosomal RNA or ITS) has been used for the majority of NGS bioaerosol studies to date (Archer et al., 2020; Barberán et al., 2015; Bottos et al., 2014; Smith et al., 2018; Tanaka et al., 2020; Woo et al., 2013). However, bioaerosol metagenomic and transcriptomic techniques have been developed (Jiang et al., 2015) and successfully applied in a handful of very recent studies. (Amato et al., 2019; Amato et al., 2017; Jaing et al., 2020). Jaing et al. (2020) performed metagenomic sequencing on bioaerosols above the Sierra Nevada mountains in the US and obtained 5,000,000 reads, although only 640,000 were successfully assigned at the genus level. Transcriptomics on microbial communities in clouds has been performed for rRNA genes (Amato et al., 2017) and untargeted amplification has been applied to full cloud metagenomes and transcriptomes to compensate for low biomass (Amato et al., 2019). These omics approaches represent the cutting edge of NGS based bioaerosol research and this study focused on a process for method optimization for the frequently used 16S and ITS amplicons.

The information produced by single amplicon NGS and conclusions drawn are sensitive to choices made when performing bioinformatic processing (Edgar, 2013, 2017). A constantly evolving multitude of environments and tools exist to process 16S or ITS NGS data (Callahan, 2020; Edgar, 2010, 2013). A typical dataset contains millions of reads, and thousands of unique sequences (amplicon sequence variants or ASVs). Sequencing errors and artefacts, even if they occur at low rates, can become significant over large datasets (Edgar, 2013). There is total reliance on automated processing and algorithms which make assumptions and introduce bias (Callahan et al., 2016). It is very difficult to objectively verify the results and determine the “true” aero-microbiome. If the environment is well understood, mock community sequencing can be performed alongside experimental samples (Hermans, Buckley & Lear, 2018). For less well understood environments like the aerosphere, more surety can be gained where results from multiple bioinformatics approaches converge. Choosing amongst the plethora of data processing options at each stage can become challenging. Especially in somewhat specialized fields, with unusual data characteristics, such as the study of bioaerosols, standard pipelines or assumptions about data can be hazardous. For instance, most decontamination pipelines assume relativity low levels of contaminating DNA compared to target DNA, which is not necessarily the case for the aero-microbiome (Pearce et al., 2016). Further, when tools are constantly being updated and “best practice” in an area can be outdated or undefined, method selection is not necessarily straightforward. An approach to select an optimal pipeline for specialized datasets, using defined selection criteria, is critically useful in the challenge to extract signal out of noise in NGS data.

### Bioinformatic data processing

**Figure 1:**
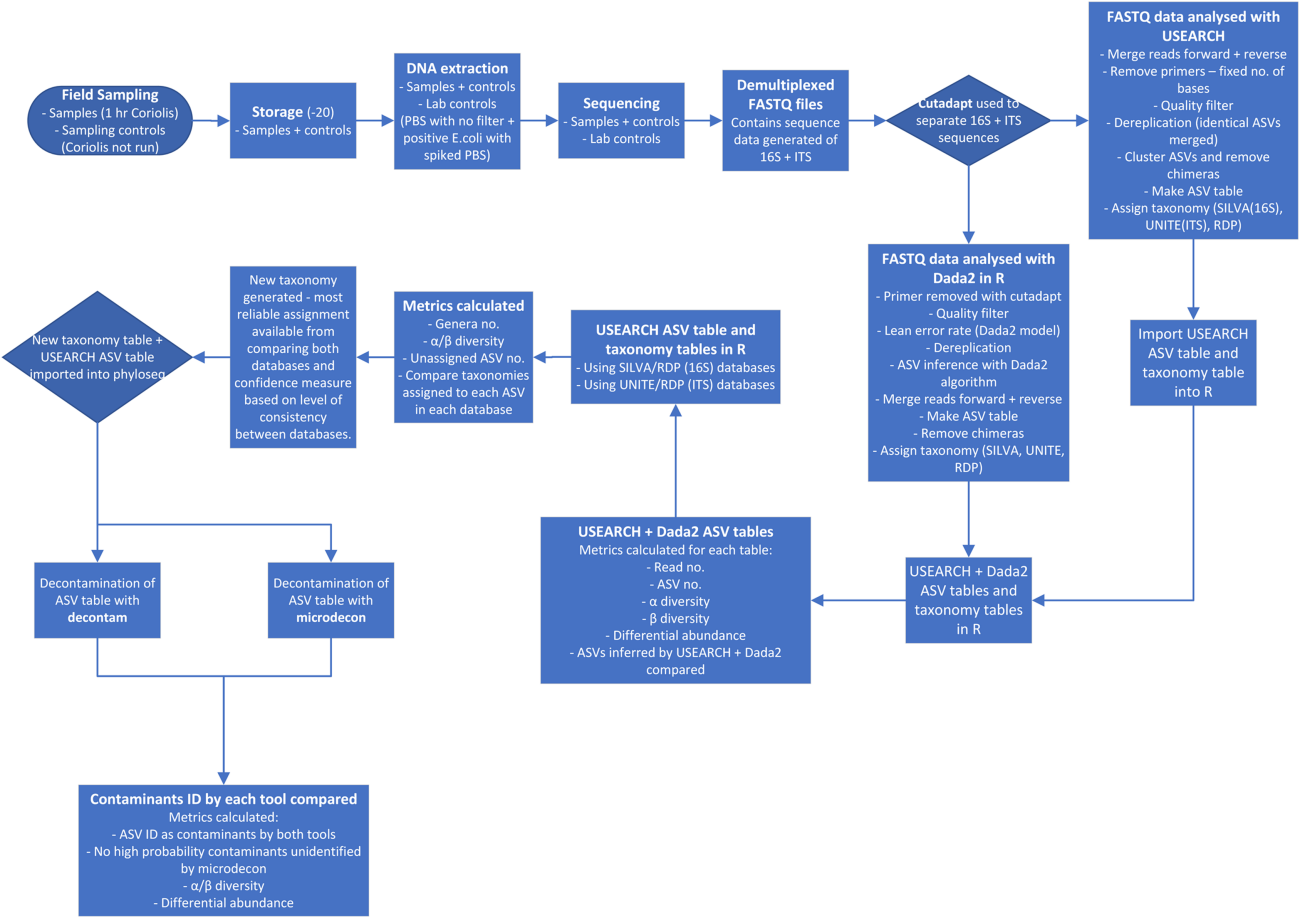
Process flow for sampling and data analysis of microbial aerosol communities at urban parks. Hr is hour. PBS is phosphate buffered saline. *E. coli* is *Escherichia coli*. No. is number. ID is identified.

Processing of 16S and ITS NGS data is a complex, multi-stage procedure and is further complicated by the necessary choices among many alternative tools available (Edgar, 2017) (Fig. 1). With so many modifications being made to the data, it is crucial to ensure each step is improving quality rather than introducing bias or errors. The key stages of the process are summarized in Fig. 1 and begin with sampling, DNA extraction, amplification of target genes and sequencing of PCR products. The sequence data is processed by separating amplicons using their respective primer sequences, trimming to remove primers, merging forward and reverse reads, quality filtering and denoising (ASV inference). Chimeras and low abundance sequences are removed. An ASV table is constructed and taxonomy is predicted with reference to sequence databases (Callahan et al., 2016; Edgar, 2017). Finally, contaminants should be filtered out of the dataset (Davis et al., 2018; McKnight et al., 2019).

Inferring the presence of the true sequence variants requires care. Approximately 0.5% (Mardis, 2013) to 0.24% (Pfeiffer et al., 2018) of base calls during Illumina sequencing are incorrect. This means that for the V3 to V4 region of the prokaryotic 16S gene, which is around 450 nucleotides long, each sequence could be expected to have two wrong nucleotides. As there is only one correct sequence but many incorrect versions as base call errors are random (Edgar, 2013), the correct variant is present at high abundance in sequence data, with many incorrect versions of the same sequence at low abundance. Most processing pipelines attempt to filter out low abundance sequences to try to remove these spurious ASVs, but filtering risks removing true low abundance organisms as well. Other sequencing artefacts, like chimeras and cross-talk (incorrect sample bar codes causing sequences to be assigned to the wrong sample) can also introduce error (Callahan et al., 2016). Comparative studies show that some tools produce many more spurious variants than others (Edgar, 2017). Two of the most popular tools currently used in bioaerosol research (Archer et al., 2020, 2019) are “Dada2” in R (Callahan et al., 2016) and USEARCH (Edgar, 2010). The use of ASVs has been recommended for bioaerosol studies, as this helps increase the resolution of the data produced (Archer et al., 2019). Operational taxonomic units (or OTUs, normally matched at 97% sequence identity) reduce the information quality compared to ASVs but lower the chance of spurious variants being detected, as low abundance incorrect variants are merged into higher abundance OTUs (Callahan et al., 2016).

The taxonomy database selected can significantly impact the number, nature and confidence of taxonomic assignments of ASVs that can be achieved. There is a trade-off between the size and the accuracy of the databases available. Larger ones, such as SILVA (Quast et al., 2012) and Greengenes (Balvočiūtė & Huson, 2017) for 16S rRNA gene, contain many uncultivated environmental sequences with taxonomy algorithmically predicted. There are thousands of conflicts in assignment of identical sequences between SILVA and Greengenes (Edgar, 2018). It is unclear which one is right, but at least one of them must be wrong. As a result, 17% of the taxonomy annotations in these databases are estimated to be incorrect (Edgar, 2018). The smaller Ribosomal Database Project training set database (RDP) is considered to be more reliable as most taxonomy is assigned based on authoritative examination of isolate strains (Edgar, 2018). However, using a smaller database, especially with novel taxa, is more likely to result in many unassigned ASVs. In addition, this does not improve the situation for unculturable organisms.

Removal of contaminant microbial taxa is critical. In addition to the challenges presented by the aero-microbiome, limited decontamination tools are available. Contaminants (especially bacteria) are ubiquitous in many reagents (McKnight et al., 2019). Clean sampling, laboratory protocols and thorough use of negative controls help. However, reads still exist in negative controls which need to be addressed. There are two principal approaches to deal with the contamination bioinformatically. Identification and removal of entire ASVs that appear to be contaminants, for example, with the R package, “decontam” (Davis et al., 2018). Contaminating ASVs are identified in “decontam” by their higher abundance in negative controls compared to samples (prevalence method) and by their concentrations inversely correlating with sample DNA concentration (frequency method) (Davis et al., 2018). Alternatively, negative controls can be used to calculate a background contamination profile, which can then be deducted from each sample, in this case with the R package “microDecon” (McKnight et al., 2019).

The aim of this protocol development study was to define a process for optimizing the bioinformatics approach for future bioaerosol studies. To achieve this, a dataset from microbial aerosol communities in urban parks was used to systematically investigate the effects of different bioinformatics choices on the amplicon ASV table and taxonomic assignments of organisms present. An optimal approach based on this information was developed. R code for the bioinformatics pipelines investigated is presented.

## Materials & methods

### Field sampling

**Figure 2:**
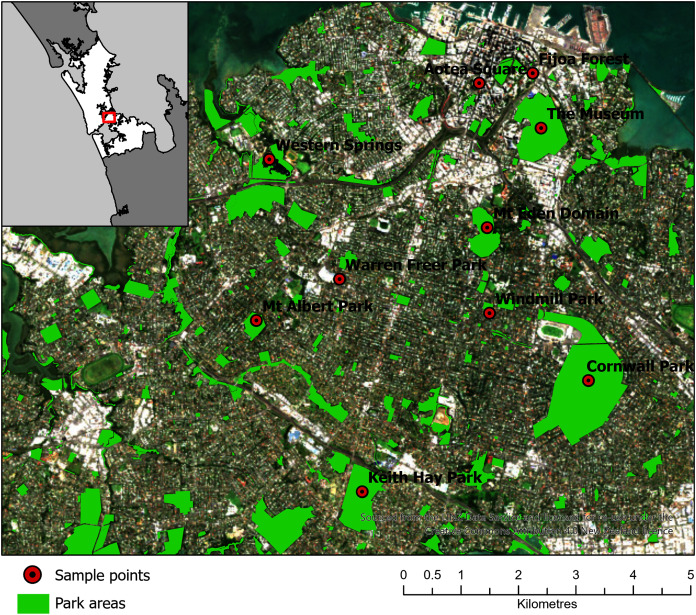
Map of 10 parks in Auckland, Aotearoa New Zealand selected for sampling in the Urban Parks study. An additional location North of Auckland is not shown. The figure was created by Bradley Case and is reproduced here with his permission.

Ten parks in urban Auckland, and a reference rural location north of Auckland, New Zealand were selected for sampling (Fig. 2). The vegetation at these parks was primarily cultivated lawns, with variable cover of deciduous and coniferous trees, shrubs and ferns. Some parks had bodies of water in them, such as Western Springs. The parks differed in occurrences of pedestrians, rates of vehicular movements and the abundance of birds and dogs. Livestock, such as sheep and cattle, were present at some. The parks differed markedly in area and altitude, as several encompassed volcanic cones. Each park was visited three times in the first sampling window (12 July 2017 to 10 August 2017) and three times in the second sampling window (20 of March 2018 to 1 June 2018). On different days during the sampling window, a single sampling location within each park was visited in a randomized order between 10 am and 4 pm and was sampled for 1 hour (h) with the Coriolis µ liquid cyclone impinger (Haig et al., 2016) at 300 L/m into phosphate buffered saline (PBS) at 1.8 m. The total volume of air in each sample was approximately 18 m^3^, which sits comfortably in the range of volumes (2.7–144 m^3^) used in similar bioaerosol studies (Amato et al., 2017). The sample was taken in the same location each time, as close to the center of the park as practicable and at minimal elevation (avoiding effects from change in altitude at volcanic peaks or being close to an edge confounding results). The exact location was saved on Google Maps (Google, 2021; Stein et al., 2016) and a photo was taken of the Coriolis aerosol sampling unit and location. During sampling, gloves were worn and the extender and unit neck, head and cone cleaned with bleach, ethanol and three rinses of MilliQ water (MQH_2_O). The cone was filled with 15 mL of PBS. A negative control was taken (PBS put into the cone without running the Coriolis) referred to as a “sampling control”. The Coriolis was run for 2–4 minutes (min) with MQH_2_O. The MQH_2_O was discarded and replaced with 15 mL of PBS and the Coriolis was run at 300 L/m for 1 h. The PBS in the cone was topped up to 15 mL after 30 min and sampling was completed with 10 mL of PBS remaining in the cone. Samples were transferred into a 15 mL falcon tube, transported in an insulated box with ice blocks and stored at −20 °C within 4 h. During sampling, observations of weather conditions were recorded (with a Kestrel 3000) and particle counts were taken (with an AeroTrak particle counter). If light rain occurred during sampling, the Coriolis was sheltered with an umbrella. If rain was heavy, the unit was packed up until the rain abated. While precipitation can affect bioaerosol communities (Reche et al., 2018), only 12 of the 66 samples in the example dataset were affected by precipitation, and therefore the data was considered representative for the purposes of defining a process for optimizing the bioinformatics approach for bioaerosol NGS data.

### DNA extraction

Samples were defrosted overnight at 4 °C. The liquid was passed through a 0.2 µm GTTP polycarbonate filter using a disposable sterile syringe. The filter holder was cleaned in bleach, ethanol and MQH_2_O between each sample. The liquid was discarded, and the filter processed using a cetyl trimethylammonium bromide (CTAB) based DNA extraction protocol. A positive extraction control (spiked with 100 µL *Escherichia coli* suspension in PBS) and a negative extraction control (no filter added—termed “laboratory controls”) was used for each DNA extraction run. The CTAB extraction protocol entailed placing the filter in a 2 mL nucleospin bead tube, filled with 0.2–0.4 mL of Qiagen 1.4 mm ceramic beads. A total of 270 µL of PBS (100 mM NaH_2_PO_4_) and 270 µL of SDS lysis buffer (100 mM NaCl, 500 mM Tris pH 8.0, 10% SDS) was added to the tube. Tubes were vortexed at top speed for 15 seconds (s) each, then shaken horizontally on a Vortex Genie 2 for 10 min. Samples were centrifuged at 13,200×*g* rpm (19,627 rcf) for 3 min. Eight mL of BME was added per 1 mL of CTAB buffer and vortexed. A total of 180 µl of the CTAB buffer and BME solution was added to the samples and incubated at 300 rpm at 60 °C for 30 min in a S1-300R Lab Companion shaker-incubator. The samples were centrifuged at 13,200×*g* rpm for 1 min. A total of 350 µL chloroform: isoamyl alcohol (24:1) was added, the samples were vortexed for 15 s then centrifuged at 13,200×*g* rpm for 5 min. The upper aqueous layer was transferred into a new 1.5 mL sterile Eppendorf tube. A total of 500 µL chloroform: isoamyl alcohol (24:1) was added and the samples were vortexed for 10 s, then left on a rocking bed (Life Technologies HulaMixer) for 20 min at room temperature. The samples were centrifuged at 13,200×*g* rpm for 5 min and the upper aqueous layers moved into a new 1.5 mL sterile Eppendorf tube. Ten M ammonium acetate to a final concentration of 2.5 M (an amount equal to 1/3 of tube volume) was added. The samples were mixed gently by repeated inversion (25 times) and centrifuged at 13,200×*g* rpm for 5 min. The upper layer was transferred to a new sterile Eppendorf tube and 0.5 times the tube volume of isopropyl alcohol was added. The samples were mixed by repeated inversion (20 times) then incubated at −80 °C for 48 h. The samples were centrifuged at 14,000×*g* rpm (20,817 rcf) for 20 min at 4 °C, the supernatant was discarded, leaving a pellet of DNA. The pellets were washed with 1 mL 70% ethanol (at −20 °C) and centrifuged at 14,000×*g* rpm at 4 °C for 5 min. The ethanol was removed, then the sample was recentrifuged a 14,000×*g* rpm at 4 °C for 5 min, then a 20 µL pipette used to remove remaining liquid. The pellets were dried in an Eppendorf concentrator plus for 8–12 min. DNA was re-suspended in 20 µL ultra-pure H_2_O (Invitrogen Ultra-Pure Distilled Water—DNAase, RNAase free) by pipetting up and down 25 times and scraping the pipette tip on the side of the tubes. The samples were heated at 55 °C for 10 min, vortexed for 10 s and stored at −20 °C. The DNA was quantified using Qbit (as per the manufacturer’s instructions), with 2 µl of the sample added to the assay tubes (Invitrogen, 2010) and test PCRs (using the same protocol as described for the 16S PCR below) in each batch to determine if further processing was needed before sequencing. All samples in batches that failed to amplify were purified with AMPure XP beads using the standard protocol (Jackson, 2016) and PCR reattempted to ensure they amplify. Sample filtration and DNA extraction were performed in a Biosafety cabinet (Gelman Sciences Biohazard-Protection class II) where possible.

### DNA sequencing

The bacterial and fungal community structure were measured using MiSeq DNA sequencing, using a method adapted from Maki et al. (2017). Fragments of 16S rDNA (approximately 460 bp) were amplified from the extracted gDNA by PCR using the universal 16S rDNA bacterial primers 314F and 785R (IDT Forward 5′- *TCG-TCG-GCA-GCG-TCA-GAT-GTG-TAT-AAG-AGA-CAG*-CCT-ACG-GGN-GGC-WGC-AG, IDT Reverse 16S 5′- *G-TCT-CGT-GGG-CTC-GGA-GAT-GTG-TAT-AAG-AGA-CAG-*GAC-TAC-HVG-GGT-ATC-TAA-TCC) and the internal transcribed spacer (ITS) fungal primers (ITS1 Forward 5′-*TCG-TCG-GCA-GCG-TCA-GAT-GTG-TAT-AAG-AGA-CAG*-CTT-GGT-CAT-TTA-GAG-GAA-GTA-A, ITS2 Reverse 5′-*G-TCT-CGT-GGG-CTC-GGA-GAT-GTG-TAT-AAG-AGA-CAG-*GCT-GCG-TTC-TTC-ATC-GAT-GC). Sequences generated were deposited with GenBank under the accession numbers KETC00000000 (16S) and KESQ00000000 (ITS). The section of the primer in italics is the Nextera adapter region, for binding of the primers for the indexing PCR. The remainder of the primer is for binding to the target area in the genome for amplification. The PCR amplicon sequences covered the variable regions V3 and V4 of the 16S rRNA gene and the fungal ITS1 region between the 18S and 5.8S rRNA genes. Thermal cycling on an Eppendorf vapo protect was performed under the following conditions for both 16S and ITS: initial denaturation at 95 °C for 3 min then, 35 cycles of 95 °C for 30 s, annealing at 55 °C for 30 s, and extension at 72 °C for 30 s. Finally, the sample was held at 72 °C for 5 min then the temperature was reduced to 4 °C. KAPA Hi-Fi Hot Start ReadyMix (KAPA) was used (12.5 µL), with 5 µL of each primer (at 1 mM concentration) and 5 µl of DNA in a 27.5 µL reaction. A total amount of 20 ng DNA was targeted in the PCR reaction. DNA was diluted if needed, therefore input concentration was variable and was often so low as to be unquantifiable, due to the minimal biomass in aerosol samples. Small DNA fragments were removed with AMPure XP beads, using the standard protocol (Jackson, 2016) then PCR products were quantified using Qbit, with 2 µl of the sample added to the assay tubes (Invitrogen, 2010). Dilution was performed to standardize DNA concentrations of each sample (16S at 5 ng/µL and ITS at 1 ng/µL) before samples were indexed to allow identification after sequencing. 16S and ITS rDNA in PCR products were amplified again using the indexing PCR primer pair, sequences binding to the regions in italics above with the addition of a unique 8-nucleotide barcode. Each position in the 96-well plates was coded by a unique pair of Nextera indexing primers that were consistent for 16S and ITS. Thermo-cycling on the GeneAmp PCR System 9700 was performed under the following conditions; initial denaturation at 95 °C for 3 min, then (8 cycles for 16S and 12 cycles for ITS) of 95 °C for 30 s, annealing at 55 °C for 30 s, and extension at 72 °C for 30 s. Finally, the samples were held at 72 °C for 5 min then at 4 °C. For the indexing PCR, 12.5 µL KAPA was used, 2.5 µL for each indexing primer, for ITS 2.5 µL of ultra-pure H_2_O was added and 5 µL of sample and for 16S, 5 µL of ultra-pure H_2_O and 2.5 µL of sample was used in a reaction totaling 25 µL. PCR amplicons from each sample were pooled, mixed, sub-sampled, purified with AMPure XP beads (Jackson, 2016), quantified (2 µl of the sample added to the assay tubes) (Invitrogen, 2010), tested with a Bioanalyzer (to check for presence/absence of primers and correct library size) and included in approximately equal amounts into a single sequencing run on a MiSeq Genome Sequencer (Illumina, MiSeq CA, USA) machine. The sequences obtained for each sample were demultiplexed based on the 8-nucleotide barcode in the indexing primers. Negative controls were sequenced so contamination could be identified and corrected for during data analysis.

### Data analysis

Data analysis was performed in R 3.6.3 x86_64 (R Development Core Team, 2010) and USEARCH (11.0.667_i86linux32 and 9.0.2132_i86linux64 ) (Edgar, 2010). Details of all code and packages used are in the Supplemental Materials. Demultiplexed FastQ files were generated. Cutadapt 2.6 (Martin, 2011) with python 3.6.9 (Van Rossum & Fred, 2009) was used to separate the two amplicons based on their primer sequences. 16S and ITS were analyzed with “Dada2 1.14.1” (Callahan et al., 2016) in R and USEARCH (Edgar, 2010) with recommended workflows (Callahan, 2021; Edgar, 2021). USEARCH outputs were imported into R using “RDPutils 1.4.1” (Quensen, 2018). Metrics were calculated for comparison between pipelines. These included numbers of reads, numbers of ASVs, alpha diversity, specifically Hill numbers, (Chao, Chiu & Jost, 2016) in “phyloseq 1.30.0” (McMurdie & Holmes, 2013), beta diversity (Bray–Curtis distance or BC) in “vegan 2.5.6” (Dixon, 2003) and differential abundance using “DESeq2 1.26.0” (Love, Huber & Anders, 2014).

ASVs inferred from USEARCH and Dada2 were compared. ASVs identified by USEARCH were used to compare different taxonomy databases. Consistent and conflicting taxonomic assignments were considered to identify the most appropriate strategy for the data. For 16S, RDP 16 (Balvočiūtė & Huson, 2017) and SILVA 132 (Quast et al., 2012) were used. For ITS, RDP 2 (Balvočiūtė & Huson, 2017) and UNITE UTAX02.02.2019 (Abarenkov et al., 2010) were used. Metrics were calculated for comparison of databases. The number of genera identified, alpha and beta diversity as above, and number of unassigned ASVs were determined. Statistical significance of differences in these metrics between pipelines was tested in base R using a non-parametric Mann–Whitney U test. The taxonomic assignment of each ASV with both databases was compared. A taxonomy file was created, which used classification from both databases and included a confidence measure. “High confidence” (assignments where both databases agreed at the genus level). “Medium confidence” (different assignments from each database). “Low confidence” (assignments only in one database). The RDP database annotation was used in preference for both ITS and 16S. If no RDP classification at the genus level was available, the alternative database was used.

The taxonomy table created was imported into “phyloseq” and subsequently processed with “decontam 1.6.0” (Davis et al., 2018). For comparison, the same dataset was also run with “microDecon 1.0.2” (McKnight et al., 2019) on default settings. The three main methods to identify contaminants available in “decontam” are: frequency, prevalence and combination (Davis et al., 2018). The combination method in “decontam” was chosen for comparison to “microDecon” as it considers the most data (both the frequency and prevalence methods combined) (Davis et al., 2018). The ASV table, after removal of contaminants, was compared to the contaminant ASVs identified by “decontam” to check consistency and identify if any ASVs highly likely to be contaminants remained. Metrics were calculated from both datasets: ASVs identified as contaminants by both tools, the number of high probability contaminants missed by “microDecon”, alpha and beta diversity and differential abundance, as above.

## Results

### ASV inference

**Figure 3:**
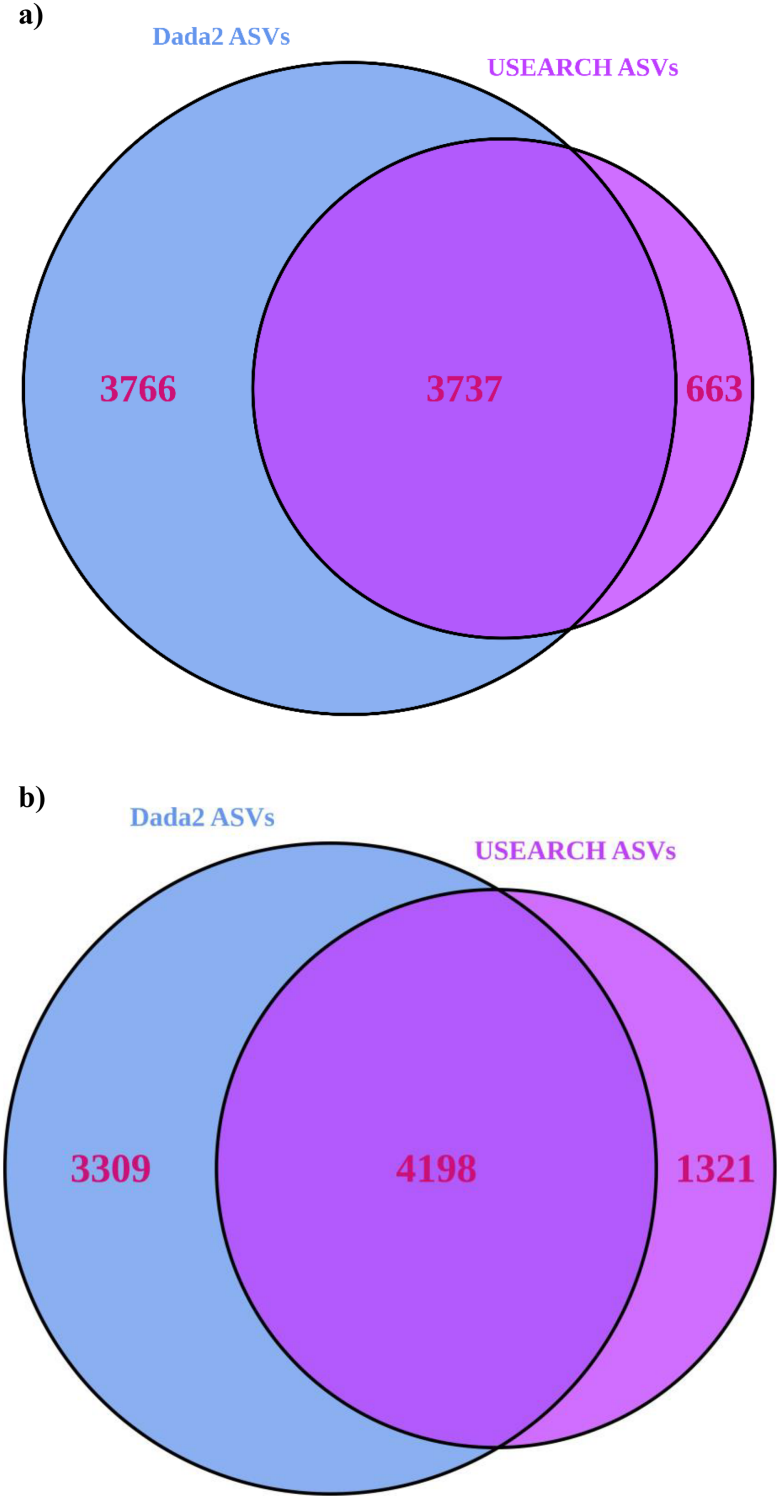
Venn diagrams showing the number of ASVs inferred from USEARCH and Dada2 for (A) 16S and (B) ITS. ASVs identified by both pipelines are very likely to be true variants.

USEARCH detected 25–33% (significantly for 16S) more reads than “Dada2” (Table 1). USEARCH was expected to return higher read counts since it matches the pre-filtered reads to ASVs if the read quality is sufficient (Edgar, 2013) while “Dada2” matches reads post-filtering (Callahan et al., 2016). More reads also meant that more samples passed through all stages in USEARCH, while in the “Dada2” pipeline several samples failed to complete the pipeline. “Dada2” consistently identified significantly many more ASVs than USEARCH, but USEARCH detected more ASVs per sample, and returned generally higher alpha and beta diversity metrics as a result. Observed alpha diversity was significantly higher for USEARCH and exponential Shannon and inverse Simpson were generally greater for USEARCH also. USEARCH data, once processed with “DESeq2”, showed slightly more ASVs were significantly differentially abundant between sample locations. Significantly differentially abundant ASVs by location were significantly higher for USEARCH. On consideration of consistency between approaches, for 16S, 50% of the “Dada2” ASVs were also present in the USEARCH data (Fig. 3). Of the ASVs inferred by USEARCH, 85% were supported by “Dada2”. A similar pattern was apparent with ITS, with 56% of “Dada2” ASVs supported by USEARCH and 76% of USEARCH ASVs supported by “Dada2”.

**Table 1:** Summary of metrics computed for datasets derived from the USEARCH and “Dada2” pipelines for ITS and 16S amplicons for microbial aerosol communities at urban parks.

		16S			ITS			
Metrics		16S Dada2	16S USEARCH	*P* value 16S Dada2 v USEARCH	ITS Dada2	ITS USEARCH		*P* value ITS Dada2 v USEARCH
Raw abundance	*Total reads*	507,586	754,752	**0.03**	1,687,012	2,222,530		0.10
	*Mean (SD) reads per sample*	9,399 (7,808)	10,938 (9,381)	0.37 (0.3)	25,179 (26,742)	33,172 (35,256)		0.09 (0.07)
Raw diversity	*Total ASVs*	7,503	4,400	**0.01**	7,507	5,519		**0.02**
Samples with no reads completing pipeline		3	0	0.08	1	0		0.36
Alpha diversity (Hill numbers)	*Observed (D0)*	151	315	**0.00**	255	441		**0.01**
	*Exp Shannon (D1)*	16.46	23.8	0.19	18.88	29.48		0.10
	*Inv Simpson (D2)*	17.25	16.66	0.90	14.8	17.15		0.61
Beta diversity (Bray–Curtis)	*Mean*	0.84	0.87	0.37	0.9	0.9		0.70
	*Median*	0.89	0.91	0.95	0.94	0.93		0.57
	*Range*	0.12–1	0.13–1	N/A	0.17–1	0.22–1		N/A
	*Standard deviation*	0.16	0.15	0.40	0.12	0.11		0.90
No. ASVs sig. differentially abundant (by location)		174	180	**0.01**	41	51		0.25

**Note:**

SD is the standard deviation. Observed (D0) is the raw diversity or number of unique ASVs detected. ExpShannon (D1) refers to the exponential of the Shannon diversity index and invSimpson (D2) refers to the inverse of the Simpson diversity index. No, is number; sig, is significantly. Ranges are not appropriate for statistical testing so an N/A appears instead of a *p* value for them. *P* values that are significant at the 0.05 threshold appear in bold.

### Taxonomic assignments

**Figure 4:**
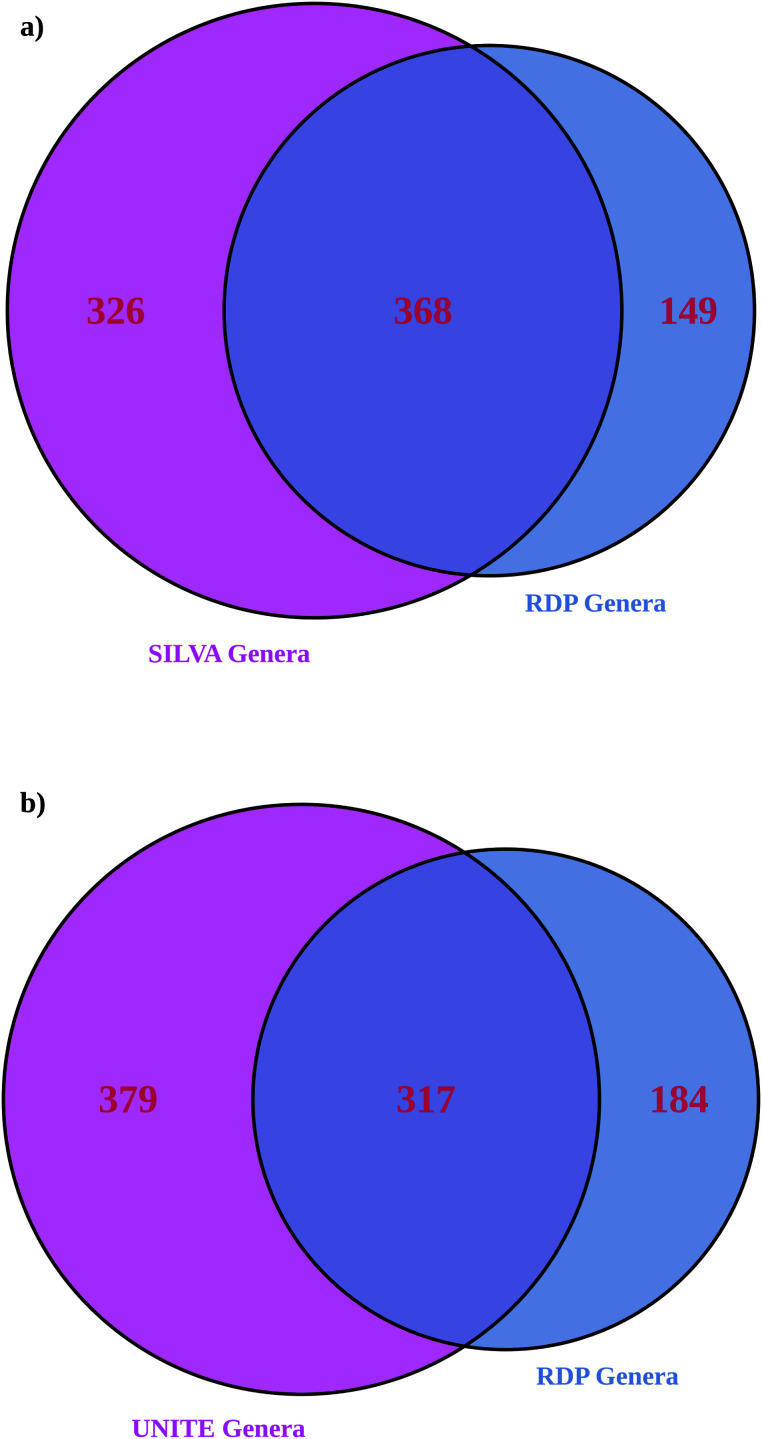
*Venn diagrams showing the number of genera identified in the RDP database only compared to* (A) *SILVA* for *16S* and (B) *UNITE* for *ITS*. This is a comparison of the total list of genera and doesn’t address how individual ASVs are mapped, which was addressed separately below.

More genera were identified with larger databases (UNITE for ITS and significantly more with SILVA for 16S), as expected (Table 2) (Edgar, 2018). 16S showed greater alpha and beta diversities with a larger database. ITS showed an inconsistent pattern of alpha and beta diversities with database size (most differences were statistically insignificant other than exponential Shannon and inverse Simpson for ITS, where UNITE was less diverse). Larger databases showed significantly fewer unassigned ASVs; RDP 16S 56% ASVs unassigned, RDP ITS 71% ASVs unassigned compared to SILVA with 38% unassigned ASVs and UNITE with 64% unassigned AS*Vs*. ITS suffered more overall from unassigned ASVs than 16S. For both amplicons, roughly half of the genera identified in the dataset matched (Fig. 4). When assignments for individual ASVs were reviewed, only 5% of 16S taxonomic assignments were consistent between RDP and SILVA at the genus level. However, for the top 100 ASVs by read count, 58% of the assignments were consistent at the individual ASV level and therefore, high confidence. For ITS, 31% of assignments were consistent between RDP and UNITE, and high confidence. When both databases were employed together to provide taxonomic information, unassigned ASVs were substantially lower for both amplicons than the largest individual database (23% for 16S and 53% for ITS ASVs remained unassigned).

**Table 2:** Comparison of taxonomy assignments of 16S and ITS USEARCH ASVs for urban parks bioaerosol communities with the RDP and SILVA (16S)/UNITE(ITS) taxonomy databases, and with a combined database approach.

Taxonomy database used		RDP training set (16S)	SILVA (16S)	*P*-value between 16S databases	RDP training set (ITS)	UNITE (ITS)	*P*-value Between ITS databases
Metrics							
Total genera identified by pipeline		517	694	**0.02**	501	696	0.06
Alpha diversity of genera (Hill numbers)	*Observed (D0)*	112	127	0.30	121	123	0.75
	*expShannon (D1)*	11.31	12.56	0.52	14	8.56	**0.02**
	*invSimpson (D2)*	7.99	8.98	0.56	8.71	5.3	**0.00**
Beta diversity of genera (Bray–Curtis)	*Mean*	0.78	0.8	0.56	0.84	0.82	0.61
	*Median*	0.8	0.83	0.52	0.86	0.85	0.75
	*Range*	0.08–1	0.09–1	NA	0.17–1	0.10–1	NA
	*Standard deviation*	0.19	0.18	0.44	0.14	0.18	0.17
Unassigned ASVs at genus level	*Number*	2,479	1,681	**0.02**	3,899	3,559	0.28
	*% total ASVs*	56%	38%	**0.00**	71%	64%	**0.01**
Unassigned ASVs at genus level with both databases	*Number*	1,011				2,871	
	*% total ASVs*	23%			53%		

**Note:**

Observed (D0) is the raw diversity or number of unique ASVs detected. ExpShannon (D1) refers to the exponential of the Shannon diversity index and invSimpson (D2) refers to the inverse of the Simpson diversity index. Ranges are not appropriate for statistical testing so an N/A appears instead of a *p* value for them. *P* values that are significant at the 0.05 threshold appear in bold.

### Decontamination

ASVs identified as contaminants were reasonably consistent between “microDecon” and “decontam”(Table 3). The large numbers of negative controls used in this study (around 1/3 of total samples) appeared useful in robust identification of contamination. 16S had 350 ASVs adjusted in “microDecon” and 344 flagged as contaminants by “decontam”. For ITS, 303 ASVs were adjusted by “microDecon” compared to 251 identified by “decontam”. Alpha and beta diversity metrics dropped slightly after processing with “microDecon”, as would be expected with removal of reads. For 16S, the number of significantly differentially abundant ASVs by location declined, while for ITS it increased slightly post decontamination.

**Table 3:** Contamination correction method tested on post USEARCH data for microbial aerosol communities at urban parks measured using 16S and ITS DNA sequencing with a combined taxonomy table.

Dataset		16S	ITS
**Metrics**			
ASVs flagged as contaminates		“microDecon” adjusted 350 ASVs for contamination. “decontam” flagged frequency—117 combination—340, prevalence 13–217 ASVs depending on threshold.	“microDecon” adjusted 303 for contamination, “decontam” flagged frequency—104 combination—251 prevalence 1–77 ASVs depending on threshold.
Alpha diversity (Hills numbers)	*Observed (D0)*	236	388
	*expShannon (D1)*	15.92	28.2
	*invSimpson (D2)*	10.45	18.03
Beta diversity (Bray–Curtis)	*Mean*	0.86	0.91
	*Median*	0.91	0.94
	*Range*	0.21–1	0.25–1
	*Standard deviation*	0.14	0.1
Number of ASVs significantly differentially abundant (by location)		144	54

**Note:**

For both ITS and 16S “microDecon” was used on default settings. The decontaminated ASV table produced was checked for consistency against contaminant ASVs identified in “decontam” on a range of its options (frequency, prevalence and combination). Observed (D0) is the raw diversity or number of unique ASVs detected. ExpShannon (D1) refers to the exponential of the Shannon diversity index and invSimpson (D2) refers to the inverse of the Simpson diversity index.

## Discussion

Bacteria and fungi observed were broadly consistent between pipelines and known to be environmental microbes or are associated with plants, humans or other animals. Fungal genera present were predominantly plant or soil associated, with many wood rotting species. Bacterial *Pseudomonas, Ralstonia* and *Methylobacterium* spp. and fungal *Penicillium, Alternaria* and *Cladosporiudum* spp. were consistent with previous bioaerosol studies (Barberán et al., 2015; Be et al., 2015; Garcia-Alcega et al., 2020). Chloroplasts were commonly observed, presumably from pollen and other plant fragments, and are abundant in similar bioaerosol studies (Brodie et al., 2007; Franzetti et al., 2010; Woo et al., 2013). Abundance and type of bacterial and fungal genera varied by location. For instance, *Bacillus* spp., which are frequently present in bioaerosols (Bottos et al., 2014), were interestingly only detected at about half of locations sampled. The results from both pipelines appeared consistent with previous bioaerosol studies.

### ASV inference

Low biomass is a particular problem for bioaerosol studies (Amato et al., 2019; Pearce et al., 2016). Therefore, preserving as many reads as possible is invaluable. The higher read counts consistently detected by USEARCH due to matching pre-filtering were preferred, since less information was lost, and raw read counts should be more accurate. Greater numbers of samples completing the pipeline for USEARCH was beneficial, since this provides more data to work with. Fewer ASVs were identified in total for USEARCH, but more per sample, such that alpha and beta diversities were generally greater than for “Dada2” (Table 1). Fewer ASVs may indicate that USEARCH is missing variants, but it may also mean that “Dada2” is detecting spurious ASVs. Greater alpha and beta diversities are likely to be desirable as more information should be available to answer the research questions posed. The number of ASVs identified as significantly differentially abundant by location was significantly higher for USEARCH, suggesting it is likely to be more informative, with the objective in mind for this dataset of detecting differences in the aero-microbiome among locations. Consistency between pipelines can offer support for the ASVs inferred, or not inferred, and indicate whether an ASV might be spurious. USEARCH ASVs appeared to be much more likely to be supported by “Dada2” than the inverse. This provided further indications that “Dada2” ASVs may be spurious. To shed light on this, read counts identified as ASVs exclusive to “Dada2” were compared to those exclusive to USEARCH. Very low abundance ASVs are likely to represent sequencing errors or artefacts, although with low biomass data genuine singletons are possible (Edgar, 2010). The more common an ASV is, the more likely it is to be a true variant. “Dada2” identified 3,766 unique 16S ASVs and just 25 had more than 100 reads in the entire dataset. A large number of very low abundance sequences made up this total. USEARCH identified 663 unique 16S ASVs and 22 of them had more than 100 reads in the entire dataset. There were also far fewer very low abundance sequences. ITS showed a similar pattern, 29 “Dada2” ASVs had over 500 reads, whereas 40 USEARCH ASVs had over 500 reads in total. Higher abundance ASVs were identified consistently by both pipelines. USEARCH appeared to be a superior choice, as it lost fewer reads, appeared to identify fewer extraneous ASVs and a much higher percentage of USEARCH ASVs were supported by “Dada2” than the inverse. On some levels the differences are minor, as most later filtering would remove all the low abundance sequences from further analysis (or they would be unlikely to impact it significantly), but the differences in diversity metrics and differentially abundant ASVs are potentially more impactful. This result demonstrates the value of assessing different pipelines for the users’ specific NGS datasets, as the recent literature typically uses “Dada2” and has moved away from USEARCH on the assumption that newer equals better (Archer et al., 2020, 2019; Mhuireach, Wilson & Johnson, 2020). While “Dada2” performs well in test community sequencing trials (Callahan et al., 2016), and is easy to implement as part of the R environment (Callahan, 2020), it doesn’t appear to be universally superior for all kinds of data. Especially when a biome diverges from the typical test community, testing a tool for the data on hand, with the specific research questions in mind, is advisable.

### Taxonomic assignments

Fewer unassigned ASVs are desirable as more inferences can be made about identified ASVs. As with ASV inference, consistency of classification between databases can lend support to the taxonomy predicted for an ASV. The trade-off between database size and accuracy is problematic (Edgar, 2018). While unassigned ASVs are disadvantageous, incorrect assignments are potentially disastrous (Edgar, 2018). Unassigned ASVs are a particular problem for fungi (Archer et al., 2020). In this dataset, ITS had up to 71% unassigned ASVs, which makes inference about the fungi difficult, but more, potentially inaccurate, assignments could lead to the wrong conclusions being drawn (Table 2). The results showed that when more genera are assigned to ASVs, alpha and beta diversity using genera generally improve, and any biological signal should be more apparent. Combining assignments from both databases was investigated to circumvent the trade-off. When information from both databases was used only 23% of 16S and 53% of ITS ASVs remained unassigned (Table 2). The process was fully automated in R to reduce error and it would be possible to add information from additional databases if more assignments and certainty were desired. With two databases, 12% to 15% more ASVs were assigned depending on amplicon, at the same or greater confidence, than in the larger database alone. A combined database approach was able to circumvent the trade-off. It performed better than classification with either database alone, and provided additional confidence information about assignments. All recent bioaerosol studies have only used a single database (Archer et al., 2020, 2019; Maki et al., 2017; Mhuireach, Wilson & Johnson, 2020; Woo et al., 2013), while suffering from a lower rate of assignments, particularly for fungi, even while using a larger and less accurate database (Archer et al., 2020). A further benefit of multi-database use arises as different databases cover different domains of life, for instance SILVA covers all three domains of life (Bowers et al., 2013). If a target region amplified is one that is shared across multiple domains, it could be beneficial to use multiple specialized databases to accurately assign as many sequences as possible, and not have to default to using the database that covers all the relevant domains. Even without using multiple databases, having an understanding of the differences in the taxonomic output when different databases are used is valuable to inform the subsequent discussion of results, and confidence placed in taxonomic conclusions, which is not the approach taken in the current literature (Archer et al., 2020, 2019; Maki et al., 2017; Mhuireach et al., 2020; Woo et al., 2013). Use, or at least consideration of, information in different taxonomic databases is suggested, as it is clear from this result that database choice can substantially alter taxonomic inference.

### Decontamination

ITS data were less contaminated overall, which is consistent with other studies (McKnight et al., 2019). ASVs identified by each approach were compared to understand the degree of differentiation between whole ASV deletion (“decontam”) or background contamination deduction (“microDecon”) approaches. At a higher level, the number of ASVs identified as potential contaminants with each tool (“microDecon” and the combination method in “decontam”) was similar (Table 3). Common ASVs, such as *Ralstonia* spp., were partially removed by “microDecon” and flagged as potential contaminants by “decontam”. These ASVs appeared to be both genuine contaminants and genuine constituents of the bioaerosol population (Waugh, Granger & Gaggar, 2010). This supports the subtraction approach in “microDecon”, as opposed to the whole ASV deletion with “decontam” for use with this dataset. There was no evidence that “microDecon” had missed contaminant ASVs. All contaminant ASVs per “decontam” with over 400 reads in the whole dataset were adjusted by “microDecon”, suggesting that no ASV deletion was required after processing with “microDecon”.

This conclusion was further tested by considering the two different types of controls that were used. Sampling controls went through the entire process and should therefore identify environmental and laboratory contamination. Laboratory controls (unused filters which went through the described protocols from DNA extraction onwards) should identify contaminating ASVs which were not present in the sampled environment. These ASVs were likely to be entirely contaminants and should therefore be deleted from the data, rather than partially subtracted. Very few ASVs in each dataset were definitive laboratory contaminants according to “decontam”. Of the few identified, they were either totally removed by “microDecon” or had under 100 total reads remaining in the whole dataset after correction. Decontamination with “microDecon” alone is likely to be the superior approach for these data, due to the cross-over between contaminating ASVs and legitimate members of the bioaerosol community and no evidence of “microDecon” omitting important contaminants. The large numbers of controls used in this study, of the two types (laboratory and sampling) proved useful in both packages for robustly identifying potential contaminating sequences, and this procedure is recommended (Davis et al., 2018; McKnight et al., 2019), particularly for the aero-microbiome or similar, where the ratio of legitimate to contaminating sequences is likely to be higher than other biomes. While ASVs were consistently identified by each package, the output ASV tables could be very different if a high abundance sequence was flagged as a contaminant and totally deleted (“decontam”) versus being partially a contaminant so that ASV was only partly adjusted (“microDecon”). Instances of this occurrence were seen in the test data used here. Therefore, these results indicate that consideration of the decontamination removal in NGS data is important and can have a large impact on the reported results and conclusions drawn. This difference is particularly marked in situations where ASVs could be both contaminants and community members, and where contaminants are prevalent. Background subtraction does not appear to be considered in the existing literature for bioaerosols, with solely the use of “decontam” appearing to be the dominant approach (Archer et al., 2020, 2019; Maki et al., 2017; Mhuireach et al., 2020; Woo et al., 2013). This work highlights the value of considering contamination correction with reference to the nature of the data set and research questions posed of it.

## Conclusions

While metagenomics and transcriptomics approaches have recently been applied to bioaerosol communities, single amplicon NGS remains the most common method used to interrogate these communities. Therefore, consideration of the process chosen to analyze single amplicon NGS data is pertinent. This study has demonstrated a methodical approach to selecting an optimized bioinformatics pipeline from a plethora of available options. Further, it has confirmed that bioinformatic data processing choices can make substantial differences in the ASV table and the taxonomy produced from 16S and ITS NGS data. Key differences were in the number of reads, ASVs inferred and ASVs remaining after decontamination. However, at a high level, datasets processed with varying combinations of approaches showed similar high abundance ASVs and taxonomic assignments. Microbiome datasets are very hard to verify, especially for poorly characterized environments like air. The utility of comparison of different approaches to sense check aero-microbiome sequence data was demonstrated. Explicit consideration of optimal approaches and use of multiple taxonomic databases, for example, were not noted in bioaerosol literature reviewed. Here, a process is laid out that researchers can step through and use to develop their own protocol, which is justified by the use of the statistics and comparisons.

### Recommendations

This study shows that understanding the characteristics of the dataset to be analyzed and choosing a bioinformatics approach using a systematic procedure is crucial for generating a high-quality dataset. For bioaerosol data, in particular, it is important to have large numbers of negative controls, with different types of controls at different times during sample collection and processing. Stringent laboratory practices are advised to reduce contamination and allow easier identification of contaminating reads in data. Comparing a wide variety of diversity metrics and outputs from multiple bioinformatics pipelines for the relevant dataset in a systematic way is recommended.

## Supplemental Information

10.7717/peerj.12065/supp-1 Supplemental Information 1 Curated code files.

10.7717/peerj.12065/supp-2 Supplemental Information 2 Raw data for Parks ITS summarized by genus and location.

10.7717/peerj.12065/supp-3 Supplemental Information 3 Raw data for Parks 16S summarized by genus and location.

10.7717/peerj.12065/supp-4 Supplemental Information 4 Complete DNA sequences for Parks ITS.

10.7717/peerj.12065/supp-5 Supplemental Information 5 Complete DNA sequences for Parks 16S.

10.7717/peerj.12065/supp-6 Supplemental Information 6 Metadata for Urban Parks. Environmental data for samples and controls taken as part of the Urban Parks study.

## Additional Information and Declarations

### Field Study Permissions

The following information was supplied relating to field study approvals (*i.e*., approving body and any reference numbers): Public parks were sampled, no permission was required to take air samples.

### DNA Deposition

The following information was supplied regarding the deposition of DNA sequences: The 16S Targeted Locus Study project is available at DDBJ/ENA/GenBank: KETC00000000. The version described in this paper is the first version, KETC01000000. The ITS Targeted Locus Study project is available at DDBJ/EMBL/GenBank: KESQ00000000. The version described in this article is the first version: KESQ01000000.

### Data Availability

The following information was supplied regarding data availability: The full R code files for all the pipelines and scorecard metrics are described in the article. The summary data for all locations for ITS and 16S and the summary for the DNA sequences for all 16S and ITS ASVs identified by USEARCH are available in the Supplemental Files.
